# Frozen Elephant Trunk Sizing: A 30,000-Feet Perspective with Thoraflex Hybrid Stent Graft

**DOI:** 10.1055/s-0042-1756667

**Published:** 2022-12-15

**Authors:** Sven Z. C. P. Tan, Idhrees Mohammed, Mohamad Bashir

**Affiliations:** 1 Barts and The London School of Medicine and Dentistry, Queen Mary University of London, London, United Kingdom; 2 Institute of Cardiac and Aortic Disorders, SRM Institutes for Medical Science (SIMS Hospital), Chennai, Tamil Nadu, India; 3 Heart Valve Disease Research Center, Rajaie Cardiovascular Medical and Research Center, Iran University of Medical Sciences, Tehran, Iran; 4 Department of Vascular and Endovascular Surgery, Velindre University National Health Service Trust, Health and Education Improvement Wales, Wales, United Kingdom

**Keywords:** frozen elephant trunk, Thoraflex Hybrid prosthesis, sizing, aorta, dissection, aneurysm

## Abstract

There is currently no standard, evidence-based approach for sizing the Thoraflex Hybrid prosthesis in frozen elephant trunk surgery. We present regional data on 906 Thoraflex grafts implanted in the United Kingdom between December 2012 and August 2021 to emphasize the heterogeneity in sizing practices and evaluate the impact this may have on clinical outcomes. Highlighting this heterogeneity will help develop an evidence-based approach to prosthesis sizing, thereby aiding decision-making for arch repair.

## Introduction

The surgical management of structural pathologies of the aortic arch and descending thoracic aorta (DTA) is associated with high rates of mortality, morbidity, and cost. Diseases of the thoracic aorta are thought to account for 1 to 2% of deaths in Western countries and are increasing in prevalence. 1

Total arch repair (TAR) is usually achieved via the frozen elephant trunk (FET) procedure. The distal stented “frozen” portion of the graft facilitates a hybrid approach to TAR, wherein the endovascular and open surgical phases can be performed concomitantly if required. 2 FET prostheses have since become the mainstay of TAR and are currently indicated in the repair of aortic arch and DTA aneurysms, acute Type A aortic dissection (ATAAD) and chronic Type A aortic dissection (CTAAD), and other pathologies such as aortic penetrating atherosclerotic ulcer (PAU), pseudoaneurysm, and intramural hematoma. 2

Several FET devices are currently on the market, including Thoraflex Hybrid (Vascutek, Glasgow, Scotland), E-Vita (CryoLife Inc., Kennesaw, GA), Cronus (MicroPort Medical, Shanghai, China), and Frozenix (Japan Lifeline, Tokyo, Japan). The Thoraflex Hybrid prosthesis (HP) has enjoyed widespread use globally, with over 931 devices used as of 2019. 2 Tan et al 2 recently published a comprehensive dataset of clinical outcomes associated with 931 Thoraflex HP implantations in an assessment of its long-term clinical efficacy, and a recent systematic review suggested that Thoraflex HP was associated with lower rates of coagulopathy-related complications than other devices. 3

The surgical approach and perioperative management for TAR with Thoraflex HP have been reported in the literature with some consensus among the aorto-vascular community. 4 5 6

Following median sternotomy and the initiation of cardiopulmonary bypass (CPB), a guidewire may be introduced retrograde into the aortic true lumen (TL) via the femoral artery to aid differentiation of the TL and false lumen (FL) after aortic resection. 4 5 6 In patients with aortic aneurysm, the HP can be deployed without the aid of a guidewire. Hypothermic circulatory arrest is established, with myocardial protection maintained via cold crystalloid or cold blood cardioplegia. 4 Adjunctive cerebral perfusion may be provided via retrograde cerebral routes or antegrade cerebral routes. 6 7 Under circulatory arrest, the diseased portion of the arch is resected, allowing inspection of the remaining DTA and identification of the TL and FL (aided by the femoral guidewire). 5 Once a graft size is selected, the graft is introduced antegrade into the DTA over the guidewire, and distal arch anastomosis is performed between the graft collar and the native aortic wall. 4 Thoraflex HP features a fourth branch allowing lower body perfusion to resume following the distal anastomosis. 2 However, in devices without this advantageous feature (such as E-vita Open Plus), a Foley catheter can be positioned in the stent graft to provide distal perfusion. 7 The proximal anastomosis can then be completed, followed by arch vessel reimplantation. The patient is then rewarmed, reperfused, and weaned from CPB.

However, even though the device's adaptability to different indications, implantation zoning, and unique features confer a great deal of versatility, there is a lack of uniformity, both regionally and internationally, over the approach to sizing the Thoraflex HP. The approach to choosing stent graft size (in terms of diameter and length) varies greatly from center to center, with varied underpinning evidence. Preoperative imaging via electrocardiogram-gated computed tomography angiography (where available) is performed to produce a multiplanar reconstruction around the aortic centerline to determine the size and location of the dissection flap, entry tears, and dimensions of the TL and FL. 8 Transesophageal echocardiography can alternatively be performed in the emergency setting. Graft size selection is based on several parameters, such as TL and FL diameter, dissection membrane elasticity, patient height, and surgical indication. 8

Sizing exerts considerable influence on overall surgical success as well as short- and long-term clinical outcomes: Idhrees et al 9 argue that narrower stent grafts make adequate sealing challenging, while wider stent grafts potentiate improper stent expansion, crumbling, intimal injury, and distal stent-induced new entry (dSINE) tears. 10 Similarly, shorter graft lengths may not fully cover the intimal entry tear, while longer grafts have been suggested to increase paraplegia risk. 9 Considering the significance of graft sizing toward patient outcomes in FET procedures, an appreciation of the heterogeneity in graft sizing practices is warranted. The objective of this observational study is to highlight the lack of uniformity in graft sizing internationally and to pave the way toward developing a standard, evidence-based approach thereto. Data on Thoraflex HP sizing in the United Kingdom are included to provide context and an appreciation of this issue on a regional level.

## Materials and Methods

Data on the Thoraflex HP use at aortic surgery centers across the United Kingdom were gathered, with a view toward contextualizing the extent of sizing heterogeneity in the aorto-vascular community. The following metrics associated with Thoraflex HP use in adult aortic surgery were included: device serial number, diameter, length, implantation date (month, year), and hospital name. Corresponding patient demographics were also gathered (age, sex, and indication). Patient identifiable information and associated clinical outcomes were excluded. These data were compiled on a Microsoft Excel spreadsheet, and frequency-based analysis was introduced to elucidate trends between prosthesis sizing, age, gender, and indication. Data on each center's case volume were also collated. The objective of data gathering for this study was to ascertain the uniformity or indeed heterogeneity of FET sizing practices in the United Kingdom, with a view toward evaluating the need for a standardized approach to FET sizing methods. Ethical review and approval were not required for this study with human participants, in accordance with the local legislation and institutional requirements.

## Results

### Patient Demographics

**Fig. 1:**
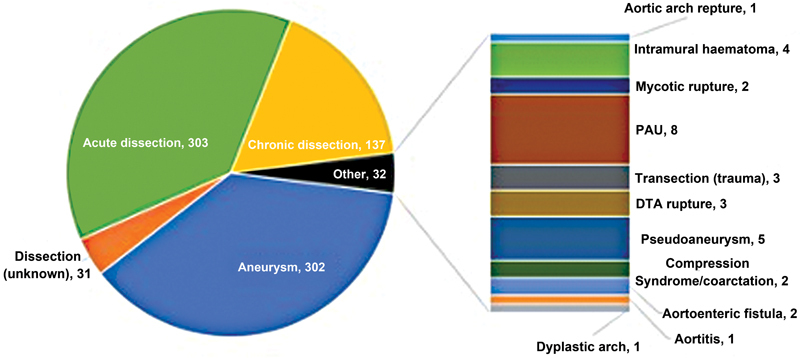
Indications for use distribution.

Data associated with a total of 906 Thoraflex HP grafts, implanted between December 2012 and August 2021, were analyzed. It is important to note that patient demographics, device size, and clinical indication information were not available for all grafts reported. Available information on patient demographics and indications for use are summarized in Tables 1 and 2 . In total, 412 grafts were known to be used in male patients and 291 in female patients. Mean age at implantation was 63 ± 13 years. Acute dissection, aneurysm, and chronic dissection were the most common indications for Thoraflex HP implantation ( *n* = 303, *n* = 302, and *n* = 137, respectively). The distribution of indication for use is depicted in Fig. 1 ( *n* = 805).

**Table 1:** Patient demographics

Characteristics	Value
Male ( *n* )	412
Female ( *n* )	291
*Age (y)* :	
Mean ± standard deviation	63 ± 13
Range	15–88
*Age breakdown (y)* :	
0–20	2
21–30	13
31–40	34
41–50	75
51–60	128
61–70	200
71–80	217
81–90	24

**Table 2:** Indication by age

	Indication										
Age range (y)	AAD	AA	AEF	CAD	CS	AD (U)	MR	PAU	Pseudo-A	AT	Total
0–20	1	–	–	–	–	–	–	–	–	1	2
21–30	7	3	2	–	–	–	–	–	–	1	13
31–40	19	7	–	6	1	–	–	–	–	–	33
41–50	38	11	–	20	–	4	–	–	–	1	74
51–60	61	25	1	30	–	7	–	–	1	–	125
61–70	48	90	–	38	–	8	2	2	2	–	190
71–80	38	130	1	33	–	7	–	2	2	–	213
81–90	3	14	–	–	–	4	–	2	–	–	23
Grand total											673

Abbreviations: AA, aortic aneurysm; AAD, acute aortic dissection; AD (U), aortic dissection (undefined); AEF, aorto-enteric fistula; AT, aortic transection; CAD, chronic aortic dissection; CS, compression syndrome; MR, mycotic rupture; PAU, penetrating aortic ulcer; Pseudo-A, pseudoaneurysm.

### Thoraflex Hybrid Prosthesis Sizing

**Fig. 2:**
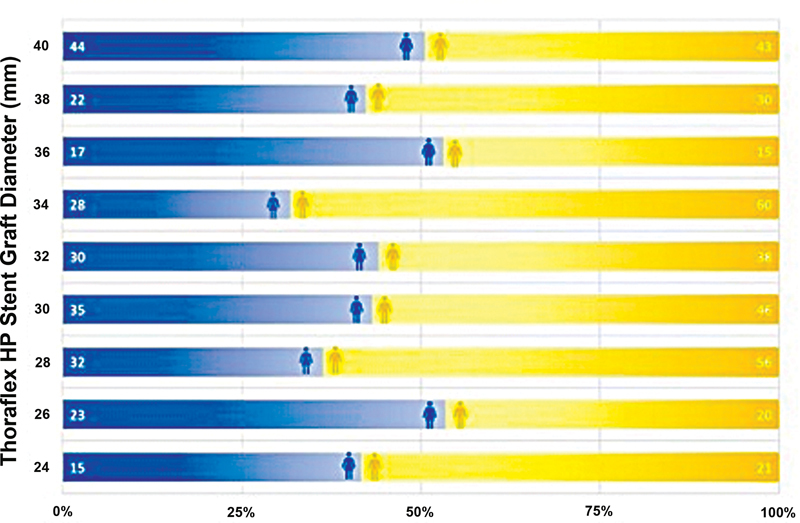
Thoraflex hybrid prosthesis diameter distribution by sex.

**Fig. 3:**
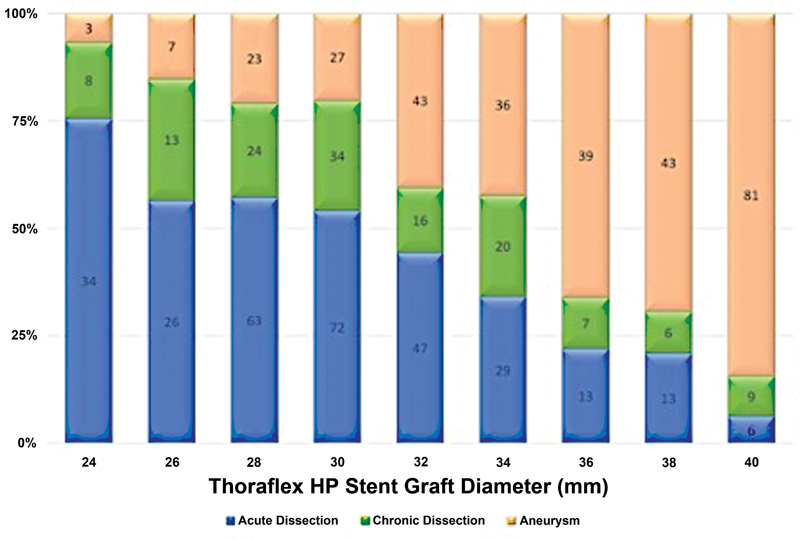
Thoraflex hybrid prosthesis diameter distribution by indication (acute dissection, chronic dissection, and aneurysm).

Device size information for 805 grafts was available. Grafts were either 22, 24, 26, 28, 30, or 32 mm in diameter and either 100 or 150 mm in length. The size distribution by patient sex is depicted in Fig. 2 , such data were available for 703 patients. Size distributions by indication ( *n* = 805) for use are depicted in Tables 3 and 4 and Fig. 3 .

**Table 3:** Thoraflex hybrid prosthesis length distribution by indication (acute dissection, chronic dissection, and aneurysm)

Indication	Stent length (mm)		Total
	100	150	
Chronic dissection	57	69	126
Acute dissection	168	100	268
Aneurysm	76	208	284
Total	301	377	678

**Table 4:** Device diameter distribution by indication (others)

	Indication											
Device diameter (mm)	AEF	CS	AD (U)	IMH	Aortitis	AC	DTAR	MR	PAU	Pseudo-A	AT	Total
24	–	–	–	–	–	–	–	–	–	–	1	1
26	–	–	1	1	–	1	–	–	–	1	1	5
28	2	1	8	−	–	–	1	1	−	2	1	16
30	–	–	5	2	–	–	–	–	1	1	–	9
32	–	–	4	–	–	–	–	–	–	–	–	4
34	–	–	7	–	–	–	–	1	1	1	–	10
36	–	–	1	–	–	–	–	–	1	–	–	2
38	–	–	2	1	–	–	1	–	2	–	–	6
40	–	–	3	–	–	–	–	–	–	–	–	7
Total	2	1	32	4	1	1	3	2	7	5	3	60

Abbreviations: AC, aortic coarctation; AD (U), aortic dissection (undefined); AEF, aorto-enteric fistula; AT, aortic transection; CS, compression syndrome; DTAR, descending thoracic aortic rupture; IMH, intramural hematoma; MR, mycotic rupture; PAU, penetrating aortic ulcer; Pseudo-A, pseudoaneurysm.

### Distribution by Region

**Fig. 4:**
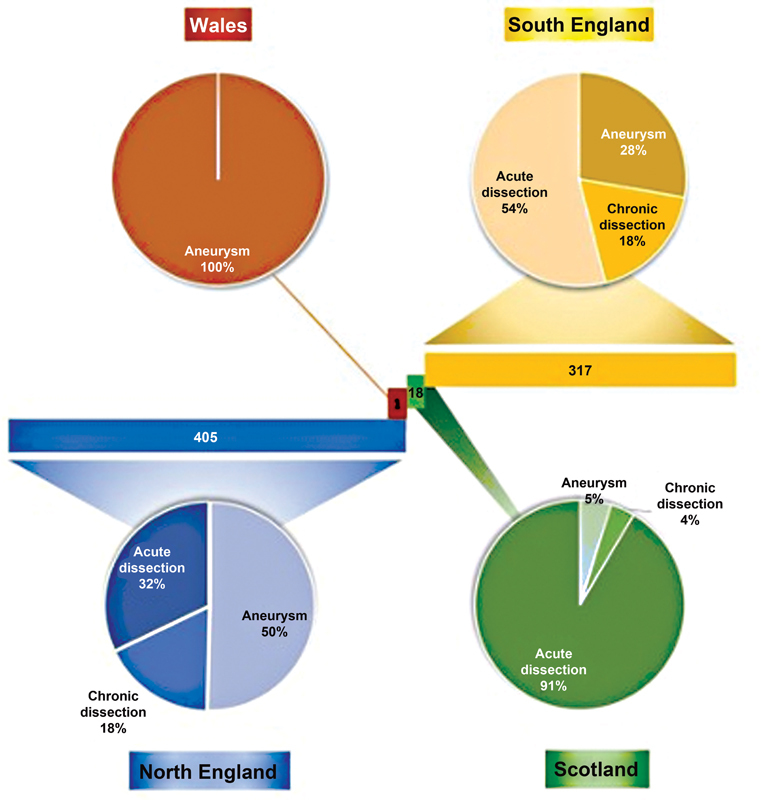
Case volume and indication for use by region.

Case volume summary by the United Kingdom region for aortic aneurysm, acute dissection, and chronic dissection are summarized in Fig. 4 . Region and indication for use were available for 741 of the grafts included.

## Discussion

### Current Approaches to Thoraflex Hybrid Prosthesis Sizing

#### Acute Type A Aortic Dissection

ATAAD accounts for a considerable proportion of patients treated with FET and is the most common indication for use in the present study ( *n* = 303, Fig. 1 ). There exist several different approaches for graft sizing in ATAAD, both within the United Kingdom and internationally. We have outlined and discussed sizing approaches to ATAAD at length in a previous study. 10

Sizing graft diameter according to the TL diameter is often recommended, as theoretically, it carries a lower risk of stent perforation. Although this method may be associated with increased endoleak and FL patency risk, thrombosis and obliteration of the FL may eventually be achieved with adequate monitoring, medical therapy, and secondary TEVAR if needed. 11 12 Tsagakis and Jakob 11 recommend against the use of oversized stent grafts in ATAAD. Rather, the maximal TL diameter at the estimated distal landing zone is used for graft diameter sizing.

In contrast, Damberg et al 8 and Katayama et al 13 suggest sizing the stent graft according to total aortic diameter at the level of the lesion. The elasticity of the dissection membrane in ATAAD allows its realignment with the adventitia, without significant aortic wall damage. Damberg et al 8 argued that using an oversized graft would therefore allow a tighter distal stent-graft anastomosis, reducing the risk of retrograde bleed.

Kreibich et al, 14 however, suggest calculating the graft diameter for ATAAD by either (1) adding 8 mm to the TL diameter at the first quarter of the dissected aorta; (2) subtracting 3 mm from the total aortic diameter between the left carotid and left axillary arteries; or (3) taking the maximal diameter of the postdissection TL.

In choosing the correct graft length, surgeons need to balance the risk of endoleak associated with short graft lengths against the potential for spinal cord injury (SCI) associated with longer grafts. 9 Adverse neurological outcomes—a familiar risk associated with aortic surgery—have been reported in cases of TAR with FET when stent grafts extending beyond T7 have been used. However, there is evidence also to suggest no association between distal landing zone and SCI incidence. 15 16 It has been suggested that longer grafts extending to T10 to 12, coupled with optimal perioperative management, are able to stabilize the aorta and mitigate the risk of residual Type B dissection and FL expansion.

#### Chronic Type A Aortic Dissection

The surgical management of CTAAD with FET is less common than that of ATAAD ( Fig. 1 ), with 137 cases of CTAAD managed with FET reported in the present analysis. With regard to graft sizing, structural differences of the chronically dissected aorta necessitate a different approach to management. This has been examined at length in a previous study. 10 Unlike in ATAAD, FL thrombosis is not always desirable—it is common for the blood supply to aortic branches (e.g., intercostal or renal arteries) to be derived from the FL. 8 Preoperative assessment of the origins of such vessels from the FL, and distal reentries thereto, is an essential step in the selection of graft diameter and length for CTAAD. Damberg et al 8 note that most surgeons avoid choosing an oversized graft so as to maintain FL patency and avoid damaging the more rigid and fibrosed dissection membrane by inflicting radial force. Kreibich et al 14 recommend using as small a diameter as possible—at most 28 mm—and note that the use of a flexible (rather than stented and rigid) elephant trunk may be useful for elective repair of CTAAD. Tsagakis and Jakob 11 similarly recommend using the smallest possible graft for CTAAD repair.

#### Aortic Aneurysm

Aortic aneurysms represent a third of cases included in the present study ( *n* = 302). The data presented in Table 3 and Fig. 3 depict a different trend in stent graft sizing for the treatment aortic aneurysm from that for dissection—stent grafts with greater diameters are favored over narrower alternatives. This is reflected in the literature, as well as being discussed at length in a previous publication; concerns over the increased risk of Type 1b endoleak associated with DTA or thoracoabdominal aneurysms means that stent graft diameter should be chosen according to the aortic diameter at the distal landing zone, with oversizing by 10–25% recommended. 8 10 11 17 Thoraflex HP instructions for use also advise oversizing stent diameter by 15 to 25% in thoracic aneurysm, with at least 40 mm of distal seal. E-Vita Open, however, does not provide any such recommendation, which may be a reason behind heterogeneity in sizing practices. 17 Oversizing graft diameter has the added benefit of making easier a later open distal anastomosis between the stent graft and what can often be a calcified, sclerotic aortic wall. The risk of Type 1b endoleak can be further mitigated by ensuring that stent graft length is sufficient to facilitate secondary thoracic endovascular aortic repair (TEVAR) for thoracoabdominal aortic stability. 8 This approach is indeed reflected by data depicted in Table 3 . Kandola et al 17 described 10% diameter oversizing with sufficient length to seal >30 mm of healthy aorta, sufficient to maintain TL patency and facilitate downstream interventions.

### What Impact Could Sizing have on Outcomes?

#### Endoleak and Reintervention

Endoleak around the distal graft in thoracic aortic dissection is a problematic, but preventable, complication that necessitates reintervention to prevent aortic rupture. 17 It is estimated that 11 to 33% of patients are affected by endoleak and require reintervention to prevent FL reperfusion and progressive aortic growth. 17 The incidence of endoleak and the requirement for reintervention associated with 931 Thoraflex HP devices have been discussed at length in a previous study. 2 Excessive FET graft oozing with E-Vita Open NEO has also been identified as a cause for reintervention, although this has not been reported with Thoraflex HP. 18 Czerny et al 19 suggest that structural mismatch between the fragile dissection membrane and the rigid stent graft is a leading cause of dSINE, and for that reason, stent graft oversizing should be avoided. Perhaps such a strategy would be appropriate for patients with fragile dissection membranes; however, in cases where the dissection membrane is deemed to be sufficiently elastic and compatible with reapproximation to the adventitia, an oversized stent graft may be advantageous. Thoraflex HP is available in varying combinations of distal stent graft and proximal graft diameters (unlike other available devices), which may be useful in cases where dissection membrane elasticity is difficult to anticipate. 17 Damberg et al 8 point out that such an approach is safe and efficacious, and indeed, implantation of an undersized stent graft may compromise optimal sealing and negate the benefits of the stented elephant trunk (compared with cET). Stent graft oversizing, especially in cases where Type 1b endoleak is anticipated (e.g., CTAAD and thoracic aneurysm), has been recommended, and average distal stent diameters of >40 mm (far greater than those reported in the present study) have been reported. 9 Such a strategy, Idhrees et al 9 argue, requires caution, later anastomosis of an oversized graft to a smaller TL is challenging. Also, although oversizing the endograft may protect against endoleak, oversizing increases the risk of the graft crumpling, producing a coarctation-like phenomenon. Kandola et al 17 also recommend oversizing as an approach to prevent endoleak development for aortic aneurysm. They report that patients affected by endoleak, or aneurysmal expansion, were treated with narrower and shorter stents compared with those without ( *p* = 0.0023 and *p* = 0.0005, respectively). In total, 3 of the 14 patients with insufficient oversizing or seal length, then, required secondary TEVAR during the follow-up period. 17 Similarly, Chu et al 20 reported zero instances of Type 1b endoleak in a case series of 40 patients undergoing FET with an oversized stent graft and 30- to 40-mm seal in the thoracic aorta. Di Bartolomeo et al 21 also noted excellent results on postoperative CTA following intervention with Thoraflex HP.

Yet, this is not to say that stent graft oversizing is not without risk. As stated above, the propensity for oversized grafts to crumple or kink is omnipresent, and Toyama et al 22 report an incidence of aneurysmal rupture post-FET due to graft kinking and subsequent endoleak. A recent systematic review also highlighted coagulopathy as a major reason for reintervention, although this seemed to affect E-Vita and Cronus devices and not Thoraflex HP and Frozenix. 3

Indeed, evidence on the relationship between FET stent graft sizing and endoleak risk is varied and requires further investigation. It is worth noting that presently, the vascular domain of EACTS does not mention or recommend an approach to determining appropriate distal stent sizing to tackle endoleak risk, compounding the lack of uniformity from center to center.

#### Neurological Outcomes

Postoperative neurological complications are, unfortunately, a familiar aspect of open and endovascular aortic surgery. In the context of TAR with FET, stent graft sizing is suggested to contribute to SCI risk. We explored this relationship in a previous study evaluating the long-term clinical efficacy of Thoraflex HP and in a previous international study on institutional FET sizing practices. 2 10 It is thought that more distal landing zones, for example, possibly augment the risk of SCI due to more extensive coverage of intercostal and segmental arteries supplying the spinal cord. 17 As a result, surgeons are tasked with balancing the risk of endoleak or suboptimal FL thrombosis associated with shorter stent grafts versus the risk of SCI or paraplegia associated with longer prostheses. 9 It is crucial to recall, however, that perioperative management and individual patient characteristics, such as circulatory arrest durations, adjunctive perfusion method, disease severity, and proximal anastomosis zone, play a huge role in determining neurological outcomes in TAR. 4 In terms of proximal anastomosis site at zone 0 or 1, data in our present study show that the 150-mm HP is used more frequently than the 100-mm HP overall but that the 100-mm HP is used more than 150 mm in the acute setting. Over 35% of the cohort were also treated with planned TEVAR (Relay Plus, Terumo Aortic, Scotland), which was not associated with endoleak, paraplegia, or stroke. Although it has been suggested that excess stenting of the DTA increases SCI risk, deeper landing zones have been credited for enabling greater aortic stabilization. 9 17 Traditionally, surgeons have avoided stenting the DTA past T7 to avoid causing SCI, even though, as Damberg et al 8 note, some retrospective studies have shown little to no association between stent graft landing zone and SCI incidence. For example, Damberg et al 8 reported no cases of SCI in their series involving patients treated with FET extending to T10 to 12, although this finding is limited by the small cohort size ( *n* = 32). Di Bartolomeo et al 21 , using the Thoraflex HP, also reported no neurological complications in their series of patients undergoing FET. Hoffman et al 23 argue that their combination of a deep landing zone with sizing guided by total aortic diameter for ATAAD is safe and efficacious and that a more aggressive stenting approach to the thoracic aorta is not associated with increased mortality, morbidity, or SCI risk. In contrast, many centers opt for shorter prostheses for dissection repair, and simply accept the planned reintervention by way of TEVAR. 9 Undoubtedly, however, stenting beyond T12 or the celiac trunk should be avoided. Interestingly, Hoffman et al 23 argue that their more aggressive strategy, with occlusion of spinal segmental arteries down to the T10 to 12 level during FET implantation, augments spinal cord perfusion due to redistribution by collateralization. The real effect of this theory on SCI incidence is unclear. The significance of perioperative management and stable hemodynamics (perhaps facilitated by Thoraflex HP's side-arm) should not be understated. These factors arguably are more important for preventing SCI than anatomic factors. 23 Therefore, debate still exists surrounding the appropriate sizing strategy to mitigate SCI risk.

#### False Lumen Thrombosis

Thrombosis of the FL is a treatment goal in acute dissection, but not always desirable in chronic dissections, as important aortic branches are sometimes supplied by the FL. 8 Undoubtedly, stent graft sizing factors which influence aneurysmal sac expansion and endoleak risk also influence FL thrombosis. However, it is worth emphasizing that FL thrombosis and obliteration is a varied and unpredictable process and can take as long as 3 months to 1 year postoperatively. 12 Yet, residual Type B dissection following Type A dissection—a consequence of failed or inadequate FL thrombosis—is described as the most important risk factor for late mortality, morbidity, and reintervention. 19 Hoffman et al 23 note that, in their series, stent graft sizing by total aortic diameter and extending to T10 to 12 lead to full obliteration of the FL around the stent graft, with no incidences of postoperative FL patency. Such an aggressive approach in ATAAD, they feel, offers the best rates of FL obliteration. This seems reasonable, as selecting stent-graft diameter according to TL diameter, avoids damage to the dissection membrane in ATAAD and allows FL patency to endure. This has been estimated to occur in 5 to 23% of cases. 23 However, the strong radial force exerted by the stent graft may potentiate further damage to the dissection membrane, although Thoraflex HP's ring-stent technology mitigates this (and subsequently paraplegia risk). This factor was examined carefully in our study covering the long-term clinical outcomes associated with 931 Thoraflex HP devices. 2

### Does the Volume–Outcome Relationship Play a Role?

In discussing uniformity and a standardized approach to FET sizing, it is worth evaluating the role of the volume–outcome relationship for a surgeon, or indeed a cardiac center. High-volume cardiac centers have demonstrated lower mortality rates in comparison to low-volume centers, and this is especially true for the management of ATAAD. Case volume distributions by regions for acute and chronic dissection, and aortic aneurysm, are depicted in Fig. 4 . Umana-Pizano et al 24 emphasize that surgical outcomes, in ATAAD specifically, are strongly influenced by the surgeon's case volume. ATAAD patients undergoing aortic repair in the hands of low-volume aortic surgeons (less than 10 ATAADs per year) without the presence of a high-volume surgeon (more than 10 ATAADs per year) were subject to an operative mortality rate of 24%, with an odds ratio of 3.72.

In 2013, the United Kingdom Aortic Surgery group was established with the aim of redefining standards of aortic care, following the demonstration of significant regional variations in access to treatment and service organization in England. 25 Between June 2013 and October 2017, 66 patients with ATAAD underwent surgical hybrid aortic repair using the Thoraflex HP at eight different high-volume centers. Initial evaluation revealed a reduction in ATAAD mortality from 23 to 12% following service restructuring and implementation of the MDT aortic program (possibly because collaboration across different specialties augments aortic care). 25 Patients treated in high-volume centers with an MDT aortic program had 50% lower mortality risk compared with lower-volume centers. Harris et al 25 also reported a significant reduction in the duration from presentation to ATAAD diagnosis and surgical repair (by 30 and 50% respectively), leading to improved survival, following the introduction of a standardized protocol within the regional hospital network. Further analysis demonstrated wide regional variation in the volume and complexity of aortic cases undertaken in United Kingdom cardiac centers. 25 The improvement in outcomes following the implementation of the United Kingdom Aortic Surgery group is surely a testament to the benefits associated with adopting a standardized, multidisciplinary approach to aortic care. Indeed, applying this to sizing practices may be beneficial for patients for whom device versatility (in terms of indications and unique features) is paramount for clinical efficacy, immediately and in long term.

In addition, the involvement of clinicians other than aortic surgeons in preoperative planning and decision-making may also be beneficial. Kandola et al 17 found that increased contact with endovascular surgeons over the duration of their study improved collaboration and expertise sharing between them and the cardiac surgeons ultimately responsible for prosthesis sizing. Indeed, inclusion of other specialties in preoperative planning and in an MDT setting could help move decisions surrounding graft sizing toward a more evidence-based approach.

Finally, considering that heterogeneity in FET graft sizing practices is not limited to the United Kingdom, an international concerted effort toward developing consensus thereon would be beneficial. Surveying accomplished, high-volume, aortic surgeons on their own sizing practices, in conjunction with extensive national data similar to that which is presented in this study, would undoubtedly help guide and inform the entire process.

## Conclusion

In summary, this comprehensive dataset highlighted that for acute dissections, 63% of grafts used were 100 mm and the majority had a diameter of 24 to 34 mm. For chronic dissections, 55% of grafts were 150 mm, with most being 28 to 34 mm in diameter. Most aneurysms (as opposed to dissections) in the cohort were treated with longer (150 mm) and wider (38–40 mm) stent grafts. Different stent graft options offer a range of advantages and liabilities. Myriad factors influence the decision over which size stent graft is appropriate, and equally, stent graft size exerts considerable influence over immediate- and long-term clinical outcomes. Both within the United Kingdom and internationally, various “schools of thought” prevail over how the optimal diameter and length of prosthesis should be determined. Notwithstanding the fact that each case is unique, a standardized, evidence-based approach is worthy of consideration and may prove useful toward streamlining care and facilitating improved overall outcomes.
